# A Bistable Switch Mechanism for Stem Cell Domain Nucleation in the Shoot Apical Meristem

**DOI:** 10.3389/fpls.2016.00674

**Published:** 2016-05-23

**Authors:** Dorjsuren Battogtokh, John J. Tyson

**Affiliations:** ^1^Department of Theoretical Physics, The Institute of Physics and Technology, Mongolian Academy of SciencesUlaanbaatar, Mongolia; ^2^Department of Biological Sciences, Virginia Polytechnic and State UniversityBlacksburg, VA, USA

**Keywords:** minimal model of SAM, reaction-diffusion systems, Turing instability, bistability, hormone field

## Introduction

In plants, the stem cells residing in shoot apical meristems (SAM) give rise to above-ground tissues (Aichinger et al., 2012). Hence, the maintenance of stem cell niches is of central importance to a plant's continued growth and development (Fletcher and Meyerowitz, 2000; Gordon et al., 2009). For the flowering plant *Arabidopsis thaliana*, the genetic determinants of stem cell growth, division, and localization have been identified, and negative feedback between a homeodomain transcription factor, WUSCHEL (WUS), and a receptor kinase, CLAVATA (CLV), is known to play a crucial role in controlling the reservoir of stem cells in the central domain of a SAM.

The morphology of plant stems and floral organs is controlled in large part by the size and stability of SAMs, which is controlled, in turn, by spatiotemporal patterns of WUS and CLV expression in meristems. For example, loss of restrictive signals in *clv* mutants of *Arabidopsis* leads to enlargement of shoot and floral meristems, resulting in extra floral organs and club-shaped siliques (Jönsson et al., 2005). The size, localization and stability of stem cell domains should be determined, in principle, by the interactions of WUS and CLV proteins, especially by their propensities to diffuse through the domain and by the rates of the molecular reactions that control their activities. Within this paradigm, reaction-diffusion (RD) models of WUS-CLV interactions have been popular mathematical models of SAM dynamics (Jönsson et al., 2005; Hohm et al., 2010; Fujita et al., 2011). In RD models, the spontaneous generation of inhomogeneous distributions of WUS and CLV in SAM domains is usually attributed to mechanisms based on a “Turing” instability (Turing, 1952; Segel and Jackson, 1972).

The generic RD equations for spatiotemporal changes in the concentrations, *u*(*x,t*) and *v*(*x,t*), of two interacting proteins are

$$\;\frac{\partial u}{\partial t}=f(u,v)+D_{u}\frac{\partial^{2}u}{\partial x^{2}},\;\frac{\partial v}{\partial t}=g(u,v)+D_{v}\frac{\partial^{2}v}{\partial x^{2}},$$

where *f(u,v)* and *g(u,v)* are nonlinear functions describing their local chemical interactions. A unique, uniform, steady-state solution, *u*(*x*,*t*) = *u*_0_ = constant and *v*(*x*,*t*) = *v*_0_, of these equations can become unstable with respect to small, non-uniform perturbations, *u*(*x*,t) = *u*_0_ + *e*^λ*t*^·δ*u*·cos(*qx*) and *v*(*x*,t) = *v*_0_ + *e*^λ*t*^·δ*v*·cos(*qx*), δ*u* < < *u*_0_ and δ*v* < < *v*_0_. The spectra of unstable modes, λ(*q*), can be found from the characteristic equation for λ. A Turing instability appears for wavenumbers close to the critical wave-number defined by the equations, λ(*q*) ≥ 0 and λ′(*q*) = 0. The *sine qua non* for Turing patterns is the condition for diffusion coefficients: *D*_*v*_ >> *D*_*u*_, generating standing waves of wavelength *l* ≈ 2π/*q*_*crit*_ in the simulations of the RD system (Gierer and Meinhardt, 1972; Murray, 2003). At present, the diffusive lengths of CLV and WUS in SAMs have not been determined, and there is no evidence to suggest that the Turing condition (diffusivity of CLV >> diffusivity of WUS) is satisfied in the central zone of a SAM.

**Figure 1:**
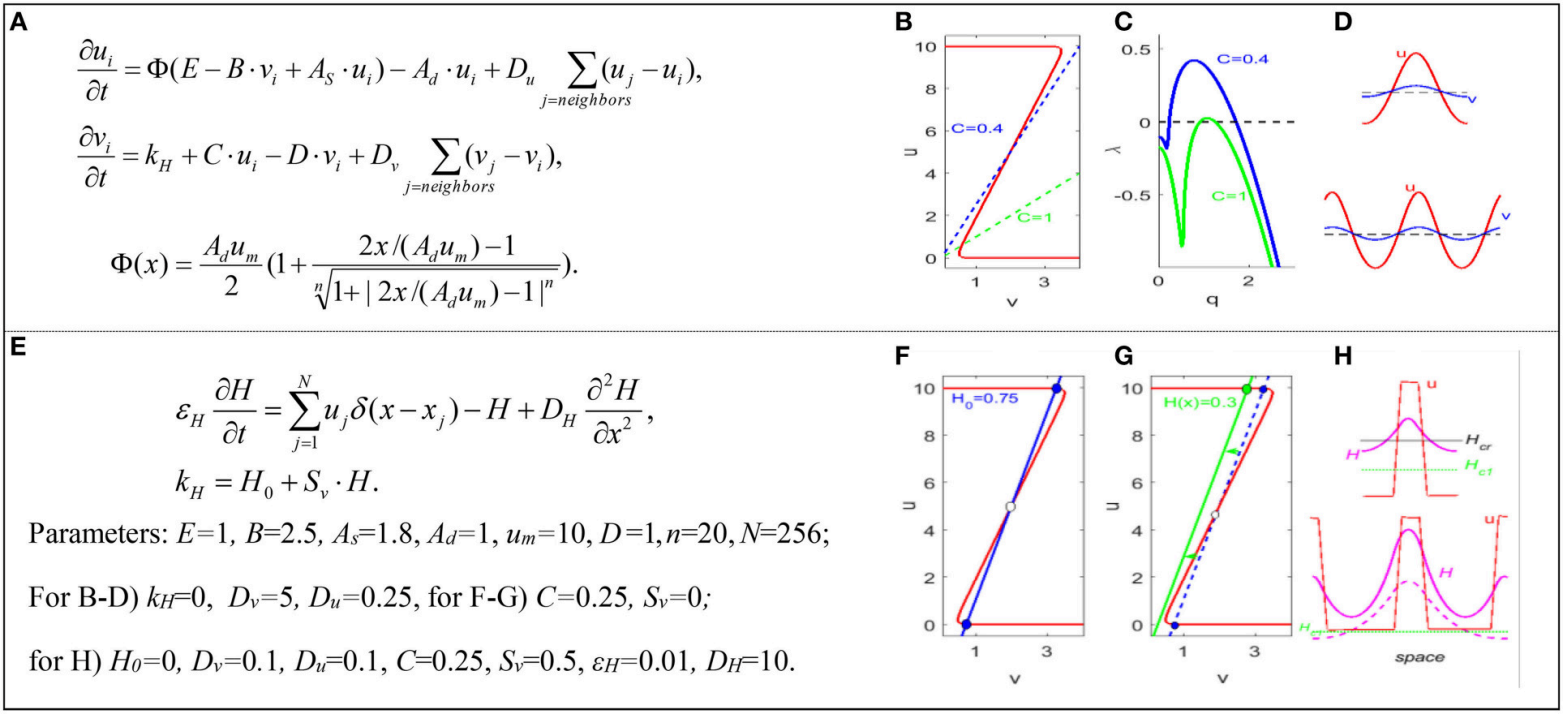
**Turing and bistable mechanisms of domain formation**. **(A)** Fujita's model of SAM. *u* = WUS, *v* = CLV, *i* = cell index, Φ is a sigmoid function. The parameters and Greek symbols are positive constants. **(B)** Nullclines. **(C)** Linear spectra of unstable modes in the continuous limit of the model. **(D)** Turing patterns (for C = 1) in smaller (top) and larger (bottom) domains. Horizontal dashed line is the uniform steady state *u*_0_ = *v*_0_. **(E)** Bistable-switch model: domain patterns are controlled by the spatial distribution of “hormone” field, *H*. *N* = number of cells, and δ = Dirac delta function. **(F)** Nullclines for a uniform *H* field. **(G)** Nullclines for a non-uniform *H* field. **(H)** Non-Turing patterns in smaller (top) and larger (bottom) domains. *Model parameters.* Function Φ describes the synthesis rate of activator *u*, with basal intensity *E*, self-activation strength *A*_*S*_, inhibitor strength *B*, and the maximum allowed value for activator, *u*_*m*_. Parameters *A*_*d*_ and *D* describe degradation rates of activator and inhibitor. Parameter *C* and *S*_*v*_ describe the intensities of inhibitor inductions by activator and hormone. *H*_0_ is the basal synthesis rate of the inhibitor. Parameter ε_*H*_ characterizes the time scale of the hormone. *D*_*u*_*, D*_*v*_, and *D*_*H*_ are diffusion coefficients. Dirac delta function describes hormone synthesis at discrete cell locations.

In typical RD models of the SAM, as in Jönsson et al. (2005), Fujita et al. (2011) and Figure 1, the interactions between WUS and CLV are described by highly nonlinear terms. Consequently, these models typically display bistability in a certain range of their parameters. Interestingly, recent experimental data on cytokinin-controlled domain confinement in SAMs (Gordon et al., 2009) suggest that WUS-CLV interactions may indeed exhibit bistability (Gordon et al., 2009).

If the differential diffusivity condition is not fulfilled, domain generation is not possible in the monostable regime of a RD system. Is spontaneous domain formation possible in the bistable regime of an RD model even if the differential diffusivity condition is not fulfilled? The answer is yes, if we supplement the RD model for WUS and CLV with a third variable describing, for example, the distribution of a rapidly diffusing hormone in the SAM region. In this brief note, we propose that the bistable-switch mechanism is superior to Turing-type mechanisms of stem-cell domain nucleation in the SAM, by comparing the two mechanisms in a minimal RD model of a SAM. We prefer the bistable mechanism because it does not require artificial assumptions, such as spatial heterogeneities for parameters and variables that typically arise in fitting Turing-type models to SAM features (Jönsson et al., 2005; Fujita et al., 2011).

## Domain nucleation mechanisms

### Turing mechanism

Figure 1A shows the activator-inhibitor model of SAM proposed by Fujita et al. (2011). The stable steady state undergoing Turing instability can be found at the intersection of the WUS(*u*) and CLV(*v*) nullclines, Figure 1B (solid and dashed lines). The linear spectrum of unstable modes can be positive, λ(*q*) > 0, and give rise to Turing patterns, if the diffusion coefficient of CLV is significantly larger than the diffusion coefficient of WUS, *D*_*v*_ >> *D*_*u*_ (see Figure 1C). In the resulting Turing pattern (Figure 1D top), the stem cell domain is the region where the level of WUS exceeds its steady state value, and other SAM domains are outside this region. In a Turing model, the size of the central domain is determined by the critical wavenumber, *q*_*crit*_, corresponding to the maximum of λ(*q*) in Figure 1C. With the meristem growth, the steady state values and critical wavenumber of the Turing pattern remain the same. When the region grows to twice the original size, the number of waves in the Turing pattern doubles (Figure 1D bottom), which can be associated with the nucleation of new stem cell domains.

For our goal of accurate prediction of the size, location, and stability of stem cell domains in SAM, the Turing mechanism has certain limitations. First, in studying a dome-shaped SAM in two and three spatial dimensions, additional assumptions introducing spatial heterogeneity are required to localize the central domain at the apex of the SAM (Jönsson et al., 2005; Fujita et al., 2011). Under such assumptions, the size and location of domain patterns are no longer defined by the critical wavenumber of the Turing instability, but they are defined by artificial restrictions imposed on the model (Jönsson et al., 2005). Second, the value of the critical wavenumber, which determines the size of the stem cell domain in a Turing mechanism, may not depend on parameters in the manner dictated by experiments. For example, experiments (Aichinger et al., 2012) indicate that the size of the stem-cell domain increases significantly, with reduction in the rate of synthesis of CLV (the parameter C in Figure 1A); however, *q*_*crit*_ decreases only a little with a 2.5-fold decrease in the value of *C* (Figure 1C). Third, Turing patterns are dissipative structures subject to fluctuations depending on noise in the system, but stem-cell domains should be stable against perturbations due to mechanical pressures arising from cell growth, cell wall extension, cell division, etc. Because of these limitations we investigated whether an alternative mechanism of domain nucleation is possible in mathematical models of SAM.

### Bistable mechanism

Depending on the parameters *k*_*H*_ and *C*, the (Fujita et al., 2011) model can exhibit a bistable regime, i.e., the nullclines of WUS(*u*) and CLV(*v*) can have three intersection points, two of which are stable steady states. Let us consider a bistable regime in the model and introduce a new diffusive variable, *H*, a hypothetical, rapidly diffusing hormone (Battogtokh, 2015). For simplicity, we also assume that *H* activity depends linearly on WUS level and that the level of CLV depends linearly on *H*. We redefine the parameter *k*_*H*_ in the equation for CLV(*v*) to integrate the *H* field into the model, see Figure 1E. The bistable steady states in the modified, three-variable bistable model of (Fujita et al., 2011) for uniform *H*_0_ are shown in Figure 1F. If we consider an initial non-uniform *H* field with a minimum value in the center (or apply a strong initial perturbation to the center of the system), a domain pattern as in Figure 1H (top) can be formed. It can be shown that for certain values of uniform *H*_0_ and nonzero *D*_*u*_ and *D*_*v*_, the domain pattern is stable because the front solution is motionless (Tyson and Keener, 1988). The Turing condition *D*_*v*_ > > *D*_*u*_ is not required for the origination and maintenance of the domain. The domain is stable even if the distribution of *H* is non-uniform, as long as *H* varies close to the value *H*_*cr*_ at which the wavefront is stationary, Figure 1H (top). The size and stability of the domain can be calculated from the condition of the stationary front (Battogtokh, 2015), while the location of the domain is controlled by the spatial distribution of the *H* field.

Spontaneous generation of new domains is possible as the system grows in size. For simplicity, let us consider growth by boundary extensions, through slow addition of new elements at the boundaries. As the system grows, the front can stay motionless, and WUS and CLV levels persist near the two stable steady-state values. However, the distribution of *H* may change with growth; it will stay high at the center where WUS level is high, but *H* level may drop in regions further away from the center. Therefore, as the system grows, the local value *H* may drop below the critical value *H*_*c1*_, where there is only an upper steady state in the corresponding local system, Figure 1G (green line). Thus, the drop of *H* below *H*_*c1*_ is accompanied by the nucleation of new domains, Figure 1H (bottom, dashed red line). The distance between the domains can be calculated from the model (Battogtokh, 2015).

## Dicussion

The framework of modeling a cell population coupled by a fast diffusive field was introduced previously by Kuramoto et al. (2000). We used the framework to model SAM cells with bistable dynamics, in the case of simple linear coupling. Exploring linear and nonlinear types of coupling with respect to experimental data may identify molecular candidates for the fast diffusive peptide-hormone. We expect that the peptide-hormone may be a member of the CLE family, for which *CLV3* is one of the founding genes (Wang and Fiers, 2010; Wang et al., 2016). While in the simple three variable mathematical model considered here, the hypothetical hormone may be sufficient to drive domain nucleation, in a more realistic model (e.g., Nikolaev et al., 2007), the involvement of several members of the CLE family (e.g., CLE 19, CLE 41, etc.) and their crosstalk with phytohormones may be necessary to account for all the biological data.

We suspect that there is a close relationship between our bistable-switch mechanism and the cell-positioning mechanism in the SAM model of Nikolaev et al. (2007). However, because of the complexity of their model, bistability and front stabilization dynamics are not as apparent as in the minimal model of SAM used here.

In simulations of a lattice of hexagonal cells, representing a two-dimensional vertical section of SAM, the bistable switch mechanism can generate WUS domains at the target position near the apex; the domain size can be controlled by the synthesis rate of CLV; and the regeneration of WUS domains seen in laser-ablation experiments can be simulated (Battogtokh and Tyson, 2016).

## Conclusion

We believe that accurate multiscale models integrating mathematical descriptions of genomic, chemical, and mechanical processes involved in plant meristem growth and development will serve for predicting and simulating plant morphogenesis. A primary requirement for such a model is a quantitative description of the relationship between the size, location, and stability of the meristems, on one hand, and the plant's shape and phenotype, on the other hand. Therefore, identifying the correct underlying mechanism of spontaneous generation of stem-cell domain patterns is crucial for creating a mathematical model of plant growth. The most basic problem with the classic Turing mechanism for domain nucleation in the SAM is that there is no evidence for differential diffusivity of CLV (inhibitor: the complex of membrane receptor kinase CLV1 and its ligand CLV3) and WUS (activator: transcription factor) biomolecules, a necessary condition for Turing pattern formation. The bistable switch mechanism, on the contrary, is not restricted by the differential diffusivity condition. Cells in the SAM can generate new stem cell domains by switching between the two stable steady states of WUS, depending on the local level of a hormonal field. The nucleated domain is not a dissipative structure but a stable structure; its size, location, and stability can be determined from the properties of the front solution connecting the two stable steady states (Battogtokh, 2015).

## Author contributions

DB designed and conducted the research, wrote the manuscript. JJT designed research and wrote the manuscript.

## Funding

Financial Assistance from the Department of Biological Sciences and the College of Science at Virginia Tech is gratefully acknowledged. DB is also partially supported by a grant of the Mongolian Foundation for Science and Technology.

### Conflict of interest statement

The authors declare that the research was conducted in the absence of any commercial or financial relationships that could be construed as a potential conflict of interest.
